# A Streamlined Workflow for Purkinje Cell Labeling and High-Resolution Analyses of Dendrites and Spines in Mice

**DOI:** 10.1523/ENEURO.0455-25.2026

**Published:** 2026-08-19

**Authors:** Xinzhu Tan, Simeng Niu, Melissa Bai, Platon Megagiannis, Rahul Suresh, Elias Pilianidis, Yosuke Niibori, David R. Hampson, Thomas M. Durcan, Yang Zhou

**Affiliations:** ^1^Department of Neurology and Neurosurgery, Montreal Neurological Institute-Hospital, Faculty of Medicine and Health Sciences, McGill University, Montreal, Quebec H3A 2B4, Canada; ^2^Re:Pair Genomics Inc., Vaughan, Ontario L6A 3M2, Canada; ^3^Leslie Dan Faculty of Pharmacy, Department of Pharmaceutical Sciences, University of Toronto, Toronto, Ontario M5S 3M2, Canada; ^4^Early Drug Discovery Unit (EDDU), Montreal Neurological Institute-Hospital, McGill University, Montreal, Quebec H3A 2B4, Canada

**Keywords:** 3D reconstruction, cell labeling, cerebellum, Cre-dependent AAV, dendritic spine, dendritic tree, Dravet syndrome, mice, Purkinje cell, Sholl analysis

## Abstract

Cerebellar Purkinje cells (PCs) exhibit a unique and highly complex dendritic architecture, which plays a crucial role in integrating synaptic inputs and shaping their physiological properties. However, fine morphological investigation of PCs at the single-cell level is challenging due to the densely packed soma and extensive dendritic arborization. Here, we present stepwise procedures for the sparse labeling of PCs via a viral-mediated approach, enabling detailed two-dimensional dendritic Sholl analysis and three-dimensional spine quantification. We demonstrated high-resolution labeling of PCs through the administration of an adeno-associated virus (AAV) carrying a conditional Cre recombinase-dependent cassette (AAV, *CAG*-*FLEX-EGFP*). The AAV was delivered through an intracerebroventricular injection in *Pcp2-Cre* neonatal mice of either sex. Subsequently, we optimized two analytical pipelines to characterize dendritic complexity and spines. As a sample application, we performed analysis on the labeled PCs from anterior (I–V) and posterior (VI–IX) cerebellar lobules in mice of both sexes. In doing so, we detected an age-dependent, lobule-specific dendritic branching pattern difference. PCs from the anterior lobules exhibited a higher spine density but a smaller spine head diameter compared with those from the posterior lobules. Additionally, we examined PC morphological perturbations in a disease model of Dravet syndrome, a disorder caused by *SCN1A* haploinsufficiency. Here, we identified reduced dendritic complexity and immature spine morphology in the PCs of *Scn1a^+/−^* mice of both sexes. Thus, we provide a labeling method that is easily adaptable, efficient, robust, and well suited for examining PC morphology during cerebellar development and related pathogenic conditions.

## Significance Statement

Obtaining specific labeling of cerebellar Purkinje cells (PCs) in vivo that is compatible with high-resolution imaging and straightforward analysis of dendritic structure and fine spine features remains challenging. The present study demonstrates that combining the *Pcp2-Cre* transgenic strain and Cre-dependent *AAV-FLEX-GFP* enables sparse and well-defined labeling of PCs. Using our optimized analytical pipeline, we detected slightly different branching patterns and spine morphology between the anterior and posterior vermis. We identified dendritic atrophy and immature spine morphology in the PCs from *Scn1a^+/−^* mice. We demonstrate that our method and analytical pipeline are reliable, straightforward, and efficient. This work establishes an optimized labeling strategy and analytical toolkit for examing PCs in vivo during cerebellar development and in disease-relevant conditions.

## Introduction

Cerebellar Purkinje cells (PCs) are among the most morphologically complex cells in the vertebrate brain, including mouse and human brains. As the sole output neurons of the cerebellar cortex, PCs receive numerous excitatory synapses from the climbing fibers and parallel fibers on their dendrites. To integrate all those inputs, the dendrites are highly elaborate and possess dense amounts of spines. This dendritic complexity is frequently disrupted in neurological disorders and thus serves as a key parameter for understanding the cell biology of PCs and the related pathogenic mechanisms associated with cerebellar dysfunction (Louis et al., 2014; Cook et al., 2021; Masoli et al., 2024; Auer et al., 2025). The shape and density of PC dendritic spines, on the other hand, are critical morphological indicators of cell function and synaptic signaling (Lee et al., 2005). Consequently, quantitative analysis of dendritic arborization and spine features in animal models are widely employed in the field of cerebellum-related research. However, the high complexity and dense branching of PC dendritic arbors make such analyses challenging, especially at the single-cell level. Traditional labeling methods, such as Golgi staining, require days to weeks of incubation and processing of the tissue. The impregnation of silver chromate is stochastic and nonuniform (Rosoklija et al., 2003, 2014). Alternative approaches include the ballistic delivery of lipophilic dyes, which lacks cell-type specificity and may therefore result in random labeling of Golgi cells and basket cells, whose dendrites frequently overlap with PC dendritic trees (Gan et al., 2000; Sillitoe et al., 2008; Zhou et al., 2020). This nonselective labeling may introduce confounds in quantitative analyses of PCs. In contrast, cell-type–specific approaches, such as immunostaining or transgenic mice expressing fluorescent proteins under PC-specific promoters, label all PCs simultaneously. Such approaches are often unattainable for image segmentation, skeletonization, or 3D reconstruction due to the dense and overlapping structures of PCs. Alternative genetic approaches, such as the “Supernova” vector system, allow single-cell labeling and gene function study (Mizuno et al., 2014). This system is highly adaptable and can be applied to various targets, enabling precise control with both temporal and spatial specificity. However, achieving PC-specific targeting with Supernova requires additional vector design and the incorporation of a PC-specific promoter (Luo et al., 2016). Other methods, such as biocytin filling, allow for single-cell labeling but require technically demanding procedures, specialized equipment, and highly specialized skills.

To overcome these challenges, we present an alternative PC labeling method that utilizes the well-established Cre-loxP system. We deployed a previously characterized *BAC-Pcp2-IRES-Cre* transgenic mouse strain that expresses Cre exclusively in PCs within the cerebellum (Zhang et al., 2004). Through intracerebroventricular injections of diluted adeno-associated virus (AAV) particles (AAV*-pCAG-FLEX-EGFP-WPRE*) into *BAC-Pcp2-IRES-Cre* mouse pups, this method enables sparse, highly specific, and efficient labeling of PCs while minimizing direct invasiveness to the cerebellum. The GFP signal labels PCs robustly across different ages in mice, enabling clear visualization of dendritic spines. Using these sparsely labeled, GFP-filled PCs, we streamlined two analytical pipelines for the high-resolution morphological characterization of PC: a 2D skeletonization-based Sholl analysis using the open-source software such as Fiji and CellProfiler (Carpenter et al., 2006; Schindelin et al., 2012; Ferreira et al., 2014; Stirling et al., 2021) and a 3D reconstruction-based spine analysis using the commercially available Imaris (Swanger et al., 2011).

To demonstrate the application of our labeling method and analytical pipeline, we first quantitatively compared the PC dendritic tree and spines between the anterior cerebellar vermis (lobules I–V) and posterior vermis (lobules VI–IX). It is well known that the cerebellar cortex exhibits lobule-specific differences anatomically, functionally, and transcriptionally. The majority of the sensorimotor regions of the cerebellum are located in the anterior lobules, while the majority of cognitive regions are in the posterior lobules (Koziol et al., 2014; Hampson and Blatt, 2015). When it comes to PCs, transcriptomic mapping has revealed that anterior lobules of the mouse cerebellar vermis are enriched with the *Plcb4^+^* subpopulation of PCs, whereas the posterior vermis is populated with *Aldoc^+^* PCs. These PC subpopulations exhibit distinct transcriptional plasticity and firing patterns (Beekhof et al., 2021; Chen et al., 2022). Transcriptional and functional distinctions usually come with morphological differences. After Sholl analysis of PCs from mice aged 1, 3, and 6 months old, we identified an age-dependent distinct dendrite branching pattern between PCs from anterior and posterior lobules, with similar overall branch complexity. Dendritic spine analysis revealed a trend toward higher spine density and smaller spine heads when comparing PCs from the anterior to the posterior vermis.

To demonstrate the utility of this approach for analyzing the morphological characteristics of PCs in the disease-relevant conditions, we employ this optimized methodology in a mouse model of Dravet syndrome (DS; Han et al., 2012). DS is a severe neurodevelopmental disorder associated with refractory seizures and an elevated risk of sudden unexpected death in epilepsy (Dravet et al., 2005; Kearney, 2013; Brownstein et al., 2018). DS is caused primarily by haploinsufficiency of *SCN1A*, which encodes the alpha subunit of the voltage-gated sodium channel Nav1.1, a key regulator of neuronal excitability. In addition to exhibiting patient-relevant autistic-like behaviors, *Scn1a^+/−^* mice show reduced sodium currents in both GABAergic interneurons and PCs, which is associated with ataxia (Yu et al., 2006; Kalume et al., 2007). Using sparse labeling and quantitative analysis, we revealed reduced dendritic complexity, increased spine length, and decreased spine density in PCs from *Scn1a^+/−^* mice. These findings suggest that *Scn1a* haploinsufficiency is associated with impaired dendritic development and altered synaptic maturation in PCs, in line with previous reports of sodium channel dysfunction and altered neuronal activity.

Collectively, these findings demonstrate that our labeling method and analytical pipeline are well suited for investigating PCs and cerebellar biology in both healthy and disease-associated pathological contexts.

## Materials and Methods

### Animals

All animal procedures have been approved by the Animal Care Committee at the Montreal Neurological Institute of McGill University, in accordance with the guidelines of the Canadian Council on Animal Care. Mice were housed with 2–5 littermates per cage in a temperature- (22–24°C) and humidity-controlled (40–60%) environment under a 12 h light/dark cycle, with food and water *ad libitum*. Animals were sex-balanced across experimental groups whenever possible, with approximately equal numbers of males and females included in each group.

*BAC-Pcp2-IRES-Cre* mice were purchased from Jackson Laboratory (strain #010536) and kept in C57/BL6 background (Zhang et al., 2004). The *Scn1a^+/−^* mice on the 129S6/SvEvTac genetic background (129S Scn1atm1Kea/Mmja22; Jackson Laboratory) were crossed with *BAC-Pcp2-IRES-Cre* mice on the C57BL/6J genetic background for Cre-dependent PC labeling.

### AAV administration

To sparsely label PCs, intracerebroventricular injection was performed in mouse pups aged Postnatal Day (P)0–P2. We first perform hypothermia-induced anesthesia by placing the pups on ice covered with a layer of aluminum foil to prevent direct contact and minimize skin damage. The anesthetized pup was then settled on a stable platform. *AAV-pCAG-FLEX-EGFP-WPRE* (Addgene #51502-AAV9; 2.5 × 10^13^ vg/ml) was used either undiluted or at serial dilutions of 1:10, 1:100, and 1:200 (Oh et al., 2014). The dilution factor can be adjusted depending on the desired labeling density. For intracerebroventricular injection, the lateral ventricles of the pups were localized based on anatomical landmarks visible through the translucent neonatal skull, as described in the literature (Kim et al., 2013, 2014). We injected 1 µl of diluted AAV with 0.1% Fast Green into the lateral ventricle on both hemispheres of the pups using a Hamilton syringe. Successful injection was indicated by the appearance of a moon-shaped Fast Green dye beneath the skull. After injection, pups were kept on a heating pad until recovery and then returned to the home cage. Injected pups were monitored daily for wound condition and nursing behavior for at least 5 d. Pups were raised to P16, P19, or 1-, 3-, 6-month-old before being terminated for experimental analyses.

### Brain slice preparation

To prepare cerebellar slices for imaging, mice were transcardially perfused with PBS followed by chilled 4% PFA. The dissected cerebellum was fixed in 4% PFA overnight and then transferred to PBS solution. Using a vibratome, the cerebellum was sectioned into 50-µm-thick sagittal floating slices and mounted onto Fisherfinest microscope slides (25 × 75 × 1 mm) with VECTASHIELD Antifade Mounting Medium (VECTH1000) and Fisherbrand Cover Glasses (0.17–0.25 mm thickness). The slides were stored at 4°C until imaging.

### Whole cerebellar slice imaging

We acquired whole cerebellar slice images using a Zeiss AxioScan slide scanner equipped with Plan-Apochromat 10×/0.45 M27 objective lens. The images were adjusted for brightness and contrast to enhance visualization.

### Dendrite quantitative analysis: image acquisition

Fluorescence images for dendrite analysis were acquired using an LSM900 confocal microscope (Plan-Apochromat 20×/0.8 M27 objective lens). To capture the full PC structure, *z*-stack images were acquired at 1 µm intervals. The upper and lower limits of the *z*-stack were defined as the planes where the fluorescent signal of the cell structure was no longer in focus. Laser intensity, pinhole size, and digital gain were kept constant across all imaging sessions for all cells in the same experiment.

### Dendrite quantitative analysis: skeletonization

Skeletonization is a computational process that reduces objects in a binary image into 1-pixel-wide representations. It allows for better structural feature characterization, such as Sholl analysis. To achieve this, acquired PC *z*-stack confocal images were first projected into a single plane using the maximum intensity projection method and saved as TIFF files in Fiji (Schindelin et al., 2012). The projected TIFF images were then imported into CellProfiler, where the Enhance or Suppress Features, Threshold, and Morphological Skeleton modules were applied sequentially to generate the cell skeleton (Carpenter et al., 2006; Stirling et al., 2021). For detailed settings, the operation under “Enhance or Suppress Features module” is “Enhance,” the feature type is “Neurite,” and the enhancement method is “Tubeness.” Other parameters, including the smoothing scale, thresholding method, and threshold-correction factor, were tested in the “Test Mode” on a batch-to-batch basis to ensure optimal skeletonization. Once optimized, the same parameters were kept consistent for all images within the same experiment. The SaveImages module was applied to save the morphological skeleton as 8 bit TIFF files. The finalized batch processing workflow was carried out with the “Analyze Images” function.

### Dendrite quantitative analysis: Sholl analysis

Skeletonized images generated in CellProfiler were reimported into Fiji for Sholl analysis. The scaling of each skeleton was set to the same as the corresponding raw confocal images using the “Set Scale” function under “Analyze.” The soma of PC was manually marked as the Sholl center, and the soma and axon skeletons were removed to avoid interference with dendritic measurements. Sholl analysis was then performed using the Neuroanatomy plugin (Ferreira et al., 2014). The starting radius was set to 5 µm, the ending radius was set to 300 µm, and the step size was set to 5 µm. We then saved the resulting table containing stepwise intersection counts for subsequent statistical analysis.

### Quantitative analysis of dendritic spine: image acquisition

Fluorescence images for spine analysis were acquired using an LSM900 confocal microscope (Plan-Apochromat 63×/1.40 oil DIC M27 objective lens). To obtain high-resolution images of PC dendritic spines within a reasonable acquisition time, zoomed *z*-stack scans were performed on the proximal and distal regions of the dendritic arbor rather than imaging the entire cell. The upper and lower limits of the stack were defined as the planes where the fluorescent signal of the cell structure was no longer in focus. The voxel size was 0.033 × 0.033 × 0.300 µm. Laser intensity, pinhole size, and digital gain were kept constant across all imaging sessions for all cells.

### Quantitative analysis of dendritic spine: 3D reconstruction

The 3D reconstruction is a process that transforms morphological structures in *z*-stack images into distinct digital objects for visualization and measurements. To perform this analysis, the acquired images were first imported into Imaris and subjected to deconvolution to correct for the point spread function (PSF; Sarder and Nehorai, 2006). Images were then 3D cropped for the region of interest (ROI), and a new filament with spine was created with Imaris Filament Tracer (Swanger et al., 2011). The primary and secondary dendritic branches of PC are largely free of spines. Therefore, we defined the ROIs as the dendritic branches of tertiary order or higher that did not cross other neurites. Dendritic tracing and spine detection parameters were optimized using representative images and then applied consistently across all samples. The detailed parameters used in our experiment are as follows: The starting points of the filaments were set on the connecting point between the secondary and tertiary branches or the edge of ROI. The “seed point thinnest diameter for branch detection” was set to 1 µm, the “thinnest diameter for spine detection” was set to 0.25 µm, and the “maximum spine length for detection” was set to 2.5 µm. These parameters could be adjusted accordingly when analyzing PCs under different experimental conditions. The Filament Tracer Wizard was trained by the user by selecting “keep” or “discard” for detecting the seed points and segments, until the Wizard generates a correct detection. A 3D mesh of dendritic segments was then automatically generated by the well-trained wizard. The output meshes from Imaris were carefully validated by the researcher under 3D view. Poorly reconstructed dendritic segments, ambiguous branches, and spine detections that were incorrectly assigned to the analyzed dendrite were removed during post hoc quality control before quantification. The filament profiles including “spine density,” “spine length,” and “spine head maximum diameter” were saved. Notably, to avoid inflated statistical power from pseudoreplication and to reduce the influence of potential detection bias, values from all detected spines within each cell were averaged, and the resulting cell-level mean was used for statistical analysis.

### Statistical analysis

All statistical analyses were carried out with IBM SPSS Statistics 31.0.0.0 under the license of McGill University. To avoid pseudoreplications arising from the hierarchical structure of the dataset, linear mixed-effects models (LMM) or generalized linear mixed-effects models (GLMM) were applied as appropriate, with “MouseID” included as the subject-level random effect (Yu et al., 2022).

## Results

### Sparse labeling of mouse cerebellar PCs

**Figure 1.**
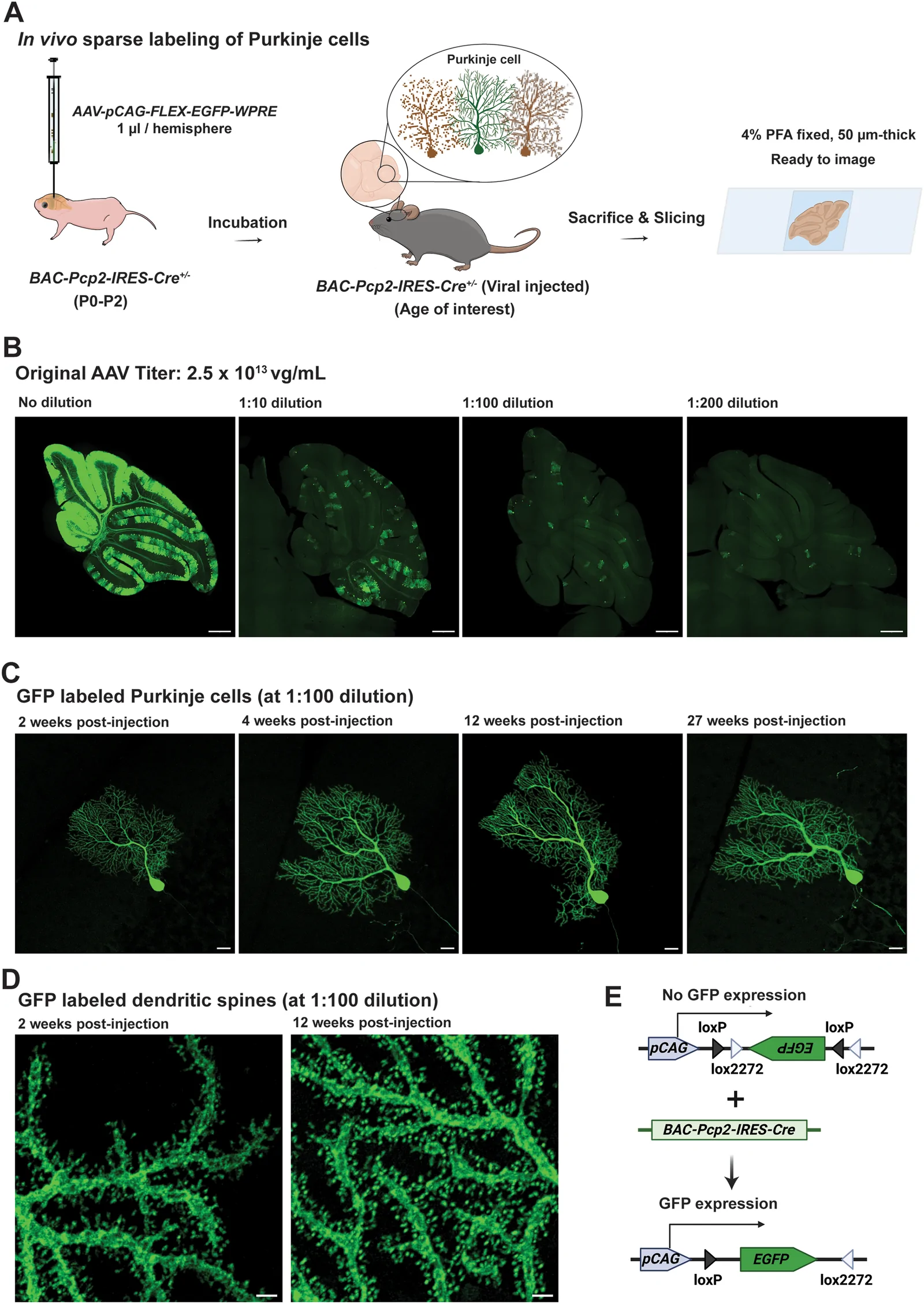
Sparse labeling of the mouse cerebellar PCs. ***A***, Schematic of the sparse PC labeling workflow. *AAV-pCAG-FLEX-EGFP-WPRE* (Addgene #51502-AAV9) is injected to mouse pups aged P0–P2, 1 µl per hemisphere in both hemispheres. Pups are raised until the age of interest, then terminated for imaging and analysis. ***B***, Whole-cerebellum fluorescent images from mice injected with different AAV titers. *AAV-pCAG-FLEX-EGFP-WPRE* (titer, 2.5 × 10¹³) was administered either undiluted or after serial dilution at the following ratios: 1:10, 1:100, and 1:200. The 1:100 dilution yielded an optimal labeling density. Scale bar, 500 µm. ***C***, Fluorescent image of individually labeled PC from mice injected with 1:100 diluted AAV at various ages. The dendritic processes are clearly visualized across all ages. Scale bar, 20 µm. ***D***, Deconvoluted high-resolution fluorescent image of the individually labeled PC from mice at 2 or 12 weeks post-injection. The dendritic spines are well resolved. Scale bar, 2 µm. ***E***, Schematic of PC-specific GFP expression through Cre-dependent switch system.

The accuracy of quantitative analysis of complex PC dendritic arbors with their densely packed spines depends on the quality of cell labeling. Here, we describe a robust method for labeling of PCs at single-cell resolution in vivo using a Cre-dependent AAV approach. We bilaterally injected *AAV-pCAG-FLEX-EGFP-WPRE* stock with the titer of 2.5 × 10^13^ vg/ml or with the following serial dilutions, 1:10, 1:100, or 1:200, into the lateral ventricles of *BAC-Pcp2-IRES-Cre* mouse pups (Fig. 1*A*,*B*,*E*). GFP expression was examined in cerebellar sections at various developmental stages. Among the tested viral concentrations, the 1:100 dilution yielded the optimal labeling density (Fig. 1*A*,*B*). We next examined the labeling efficiency across different developmental stages of interest. Following injection of the 1:100 dilution of the virus into newborn mice, labeled PCs were imaged from animals at 2, 4, 12, and 27 weeks postinjection. Using confocal imaging with a 20×/0.8 objective (pixel size 0.312 × 0.312 µm) and *z*-projection, we can clearly visualize individual PCs with lighted up dendritic trees across all ages examined, from neonatal stages to mature adulthood (Fig. 1*C*). We also performed high-magnification confocal imaging with a 63×/1.4 oil-immersion objective (pixel size 0.033 × 0.033 µm) on labeled PCs from animals at 2 and 12 weeks postinjection and observed clearly resolved dendritic spines (Fig. 1*D*).

**Figure 2.**
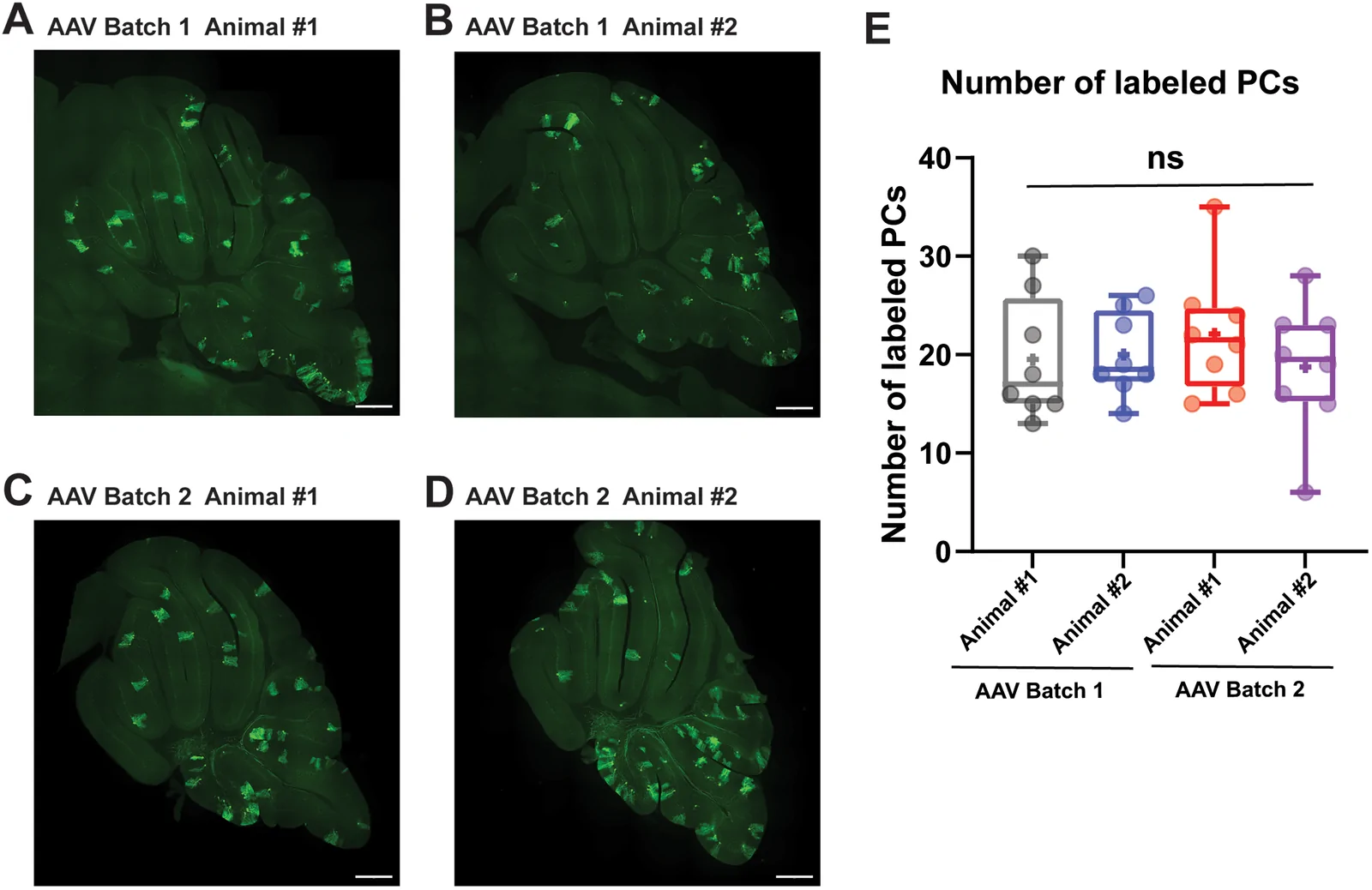
Labeling stability across animals and AAV batches. ***A***, Representative whole-cerebellum fluorescence images from Animal #1 injected with AAV from Batch 1. ***B***, Representative whole-cerebellum fluorescence images from Animal #2 injected with AAV from Batch 1. ***C***, Representative whole-cerebellum fluorescence images from Animal #1 injected with AAV from Batch 2. ***D***, Representative whole-cerebellum fluorescence images from Animal #2 injected with AAV from Batch 2. ***E***, Quantification of labeled PCs revealed no significant difference between animals and AAV batches. For each animal, eight cerebellar vermal slices were randomly selected for imaging and quantification. One-way ANOVA, *F* = 0.4803; *p* = 0.6986. Scale bar: (***A***–***D***) 500 µm. Representative serial whole-cerebellum sections from AAV-injected animals are shown in Extended Data Figure 2-1.

Next, we assessed the reproducibility of this labeling method using two separate, independently prepared AAV batches, with two animals injected with each batch. For each animal, we randomly selected eight cerebellar vermis sections for imaging. On average, ∼20 PCs per cerebellar slice were sparsely labeled with bright, nonoverlapping signals across all four animals (Fig. 2*A*,*B*; Extended Data Fig. 2-1). While the exact number and spatial distribution of labeled cells varied slightly across slices and animals, likely due to differences in transducing efficiency of AAV as well as tissue quality, including perfusion and sectioning, the overall labeling efficiency remained consistent and stable across experimental batches. These results demonstrate the suitability of this Cre-dependent, AAV-mediated sparse labeling method for quantitative morphological analysis of PCs in mice. This single-cell–level labeling method enables analysis without the complex image preprocessing typically required to extract major morphological features of individual neurons in densely labeled structures.

10.1523/ENEURO.0455-25.2026.f2-1 Figure 2-1 **Whole-cerebellum images from AAV-injected animals showing labeling stability** A. Animal #1 (female, P63) injected with AAV batch 1. B. Animal #2 (female, P63) injected with AAV batch 1. C. Animal #1 (female, P53) injected with AAV batch 2. D. Animal #2 (male, P53) injected with AAV batch 2. Scale bar: 500 µm. Download Figure 2-1, TIF file.

### Dendritic morphology analysis across developmental stages in sparsely labeled PCs

**Figure 3.**
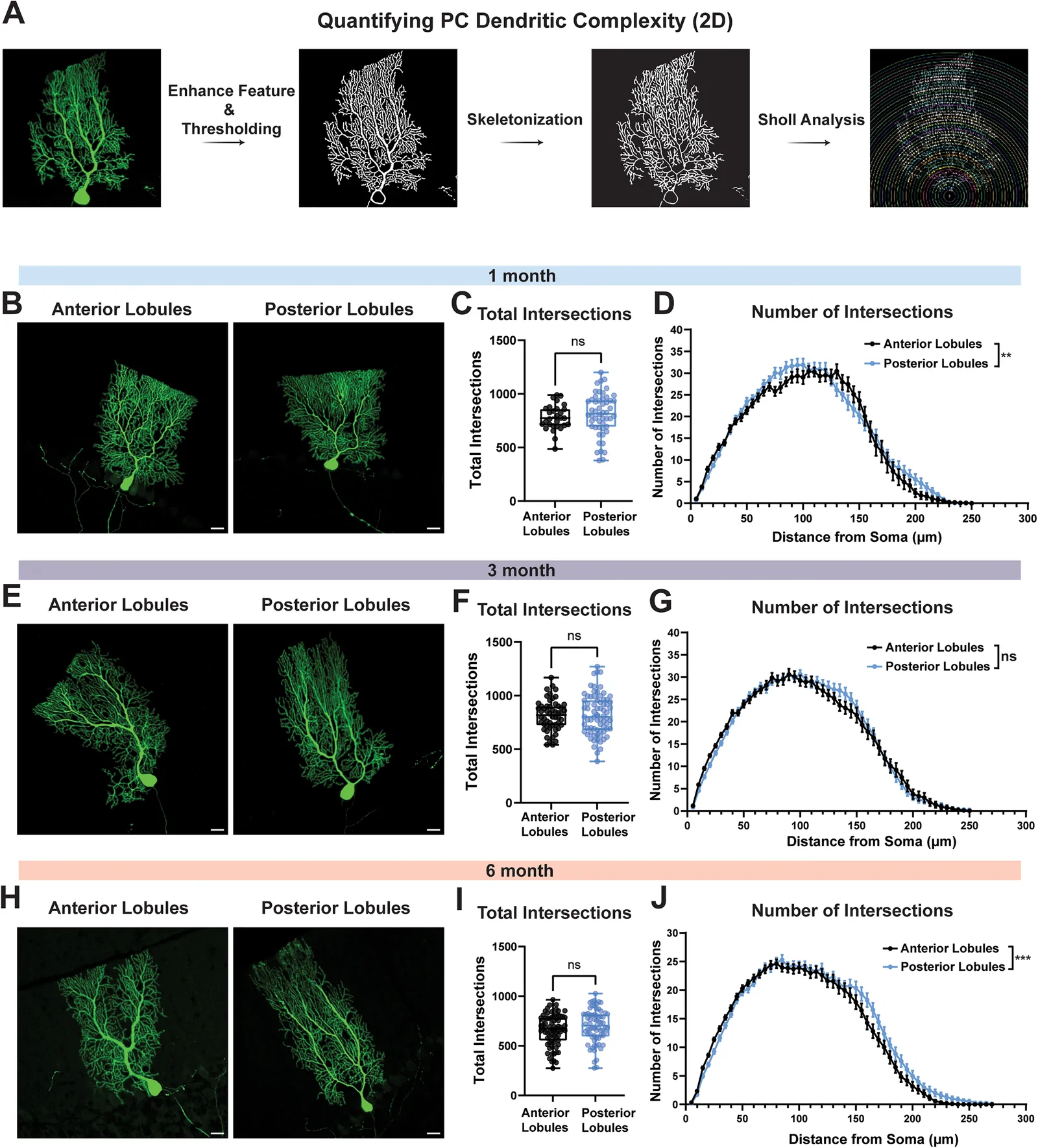
Dendritic morphology analysis at different developmental stages in sparsely labeled PCs. ***A***, Schematic of the pipeline for dendritic complexity analysis. Through CellProfiler, fluorescence images of GFP-labeled PC are subjected to enhancing the features of neurites, thresholding into binary images, and then skeletonizing into 1-pixel-wide representations. The PC skeletons were then used to perform Sholl analysis in ImageJ. ***B***, Representative fluorescence image of labeled PCs at 1 month old, from the anterior and posterior vermis. Scale bar, 20 µm. ***C***, Quantification and statistical analysis of the total intersections between skeletonized PC dendrites and the Sholl concentric circles in the 1-month-old mice. No significant difference was detected between PCs from the anterior and posterior vermis. *N* = 29 PCs for the anterior vermis and *N* = 52 PCs for the posterior vermis. All data collected from three mice (2 males, 1 female), with “MouseID” as a subject-level random effect. LMM, *F*_(1,79)_ = 0.511; *p* = 0.477. ***D***, Sholl analysis of the PC dendritic structure revealed a significant lobular difference in PCs from the anterior and posterior vermis of the 1-month-old mice. *N* = 29 PCs for the anterior vermis and *N* = 52 PCs for the posterior vermis. All data were collected from three mice (2 males, 1 female), with “MouseID” as a subject-level random effect. GLMM with a negative binomial distribution and log link: radius (*F*_(48,3950)_ = 66.538; *p* < 0.001), lobule (*F*_(1,3950)_ = 8.556; *p* = 0.003), lobule by radius interaction (*F*_(46,3950)_ = 1.458; *p* = 0.024). ***E***, Representative fluorescence image of labeled PCs at 3 months old, from the anterior and posterior vermis. Scale bar, 20 µm. ***F***, Quantification and statistical analysis of the total intersections between skeletonized PC dendrites and the Sholl concentric circles in the 3-month-old mice. No significant difference was detected between PCs from the anterior and posterior vermis. *N* = 54 PCs for the anterior vermis and *N* = 73 PCs for the posterior vermis. All data collected from three mice (2 males, 1 female), with “MouseID” as a subject-level random effect. LMM, *F*_(1,124.936)_ = 0.030; *p* = 0.862. ***G***, Sholl analysis of the PC dendritic structure did not detect a significant lobular difference in PCs from the anterior and posterior vermis of the 3-month-old mice. *N* = 54 PCs for the anterior vermis and *N* = 73 PCs for the posterior vermis. All data collected from three mice (2 males, 1 female), with “MouseID” as a subject-level random effect. GLMM analysis (negative binomial, log link): radius (*F*_(49,6250)_ = 69.365; *p* < 0.001), lobule (*F*_(1,6250)_ = 0.012; *p* = 0.914), lobule by radius interaction (*F*_(48,6250)_ = 0.332; *p* > 0.99). ***H***, Representative fluorescence image of labeled PCs at 6 months old, from the anterior and posterior vermis. Scale bar, 20 µm. ***I***, Quantification and statistical analysis of the total intersections between skeletonized PC dendrites and the Sholl concentric circles in the 6-month-old mice. No significant difference was detected between PCs from the anterior and posterior vermis. *N* = 74 PCs for the anterior vermis and *N* = 68 PCs for the posterior vermis. All data collected from four mice (2 males, 2 females), with “MouseID” as a subject-level random effect. LMM, *F*_(1,140)_ = 1.794; *p* = 0.183. ***J***, Sholl analysis of the PC dendritic structure revealed a significant lobular difference in PCs from the anterior and posterior vermis of the 6-month-old mice. *N* = 74 PCs for the anterior vermis and *N* = 68 PCs for the posterior vermis. All data were collected from four mice (2 males, 2 females), with “MouseID” as a subject-level random effect. GLMM analysis (negative binomial, log link): radius (*F*_(53,7560)_ = 84.508; *p* < 0.001), lobule (*F*_(1,7560)_ = 55.300; *p* < 0.001), lobule by radius interaction (*F*_(47,7560)_ = 2.696; *p* < 0.001).

PCs from the anterior and posterior cerebellar lobules have been reported to exhibit heterogeneity in gene transcription and cellular function (Wu et al., 2019; Chen et al., 2022). To demonstrate the applicability of this AAV-mediated sparse labeling approach for dendritic analysis, we investigated whether there are detectable morphological differences among PCs from the anterior compared with the posterior lobules. We injected three cohorts of mice and analyzed at 1, 3, and 6 months of age. After slice preparation and imaging, PCs were skeletonized using CellProfiler, and we performed Sholl analysis through the neuroanatomy plugin of Fiji (Fig. 3*A*; Carpenter et al., 2006; Schindelin et al., 2012; Ferreira et al., 2014). We examined the difference in dendritic complexity between PCs from the anterior vermis and the posterior vermis in all three cohorts (Fig. 3*B*,*E*,*H*). No significant difference was observed in the total number of intersections across the three cohorts using LMM, indicating similar overall dendritic branching between the anterior and posterior vermis (Fig. 3*C*,*F*,*I*). However, GLMM, which account for repeated count measurements across radii, revealed a significant effect of lobule and a significant lobule by radius interaction in the 1- and 6-month-old cohorts (Fig. 3*D*,*J*), but not in the 3-month-old cohort. These findings suggest age-dependent regional differences in dendritic branching patterns between PCs in the anterior and posterior vermis (Fig. 3*G*). Together, these results indicate that our AAV-mediated high-resolution, sparse labeling method and analytic pipeline are robust and sensitive to detect even minor morphological differences among PCs.

### Three-dimensional quantitative analysis of dendritic spines in sparsely labeled PCs

**Figure 4.**
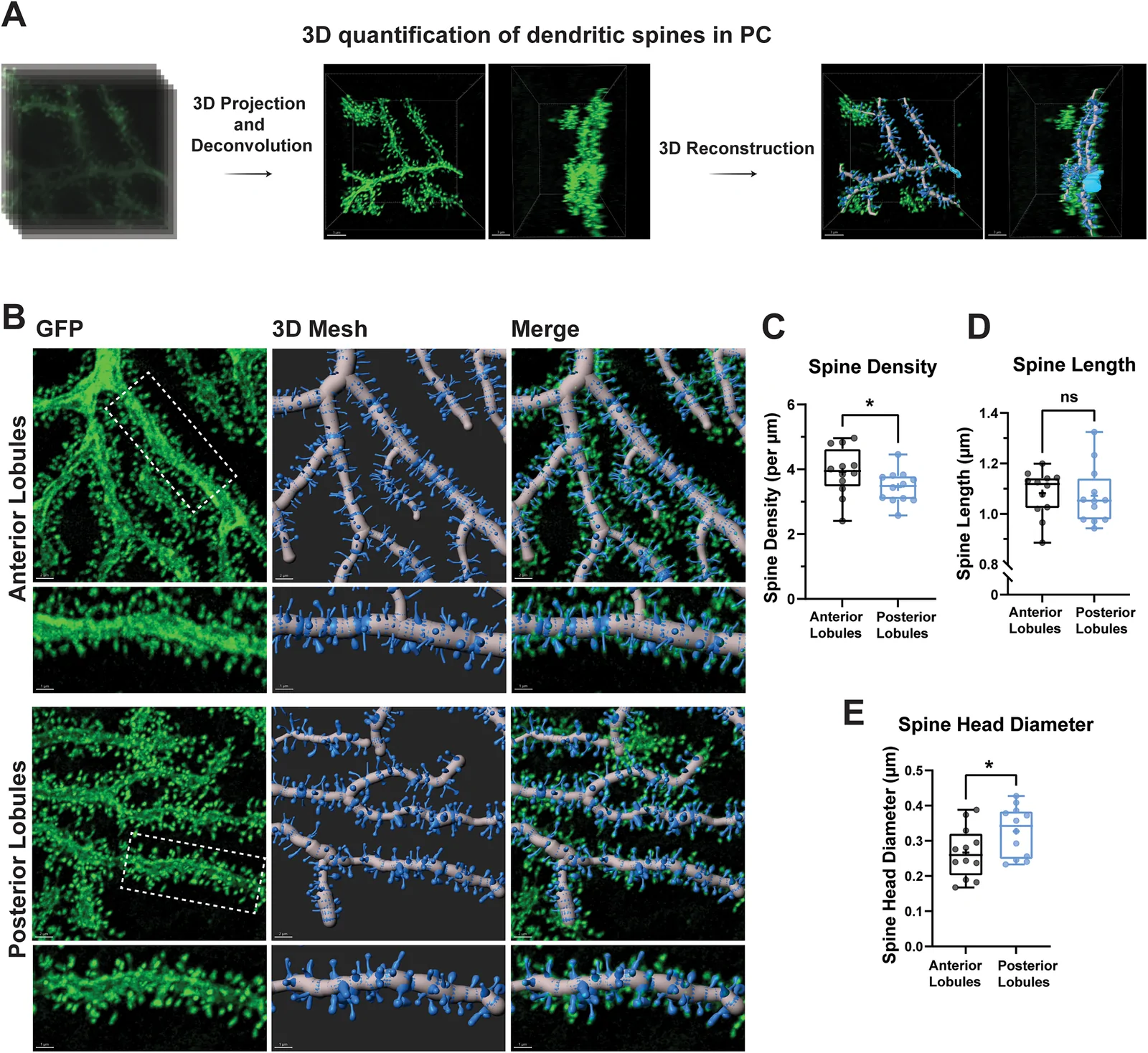
3D quantitative analysis of dendritic spines in sparsely labeled PCs. ***A***, Schematic of the pipeline for spine analysis. Through Imaris, fluorescence images of GFP-labeled PC are 3D projected, deconvoluted, and reconstructed into 3D meshes for visualization and measurements. ***B***, Representative fluorescence image and reconstructed 3D mesh of spines from PC in the anterior and posterior vermis. Scale bars: 2 µm (top panels) and 1 µm (zoomed-in bottom panels). ***C***, Statistical analysis of the spine density in PCs from anterior and posterior vermis revealed a significant difference. *N* = 12 PCs for the anterior vermis and *N* = 12 PCs for the posterior vermis. Data points from each cell were averaged as one biological replicate. All data collected from four mice (2 males, 2 females), “MouseID” as a subject-level random effect. LMM, *F*_(1,19)_ = 5.806; *p* = 0.026. ***D***, Statistical analysis of the spine length in PCs from anterior and posterior vermis did n't detect a difference. *N* = 12 PCs for the anterior vermis and *N* = 12 PCs for the posterior vermis. Data points from each cell were averaged as one biological replicate. All data collected from four mice (2 males, 2 females), “MouseID” as a subject-level random effect. LMM, *F*_(1,19)_ = 0.109; *p* = 0.744. ***E***, Statistical analysis of the spine head diameter in PCs from anterior and posterior vermis revealed a significant difference. *N* = 12 PCs for the anterior vermis and *N* = 12 PCs for the posterior vermis. Data points from each cell were averaged as one biological replicate. All data collected from four mice (2 males, 2 females), “MouseID” as a subject-level random effect. LMM, *F*_(1,19)_ = 5.467; *p* = 0.030.

To explore the potential of our sparse PC labeling method for dendritic spine analysis, we further studied the spine properties of PCs from the anterior and posterior vermis. We imaged the dendritic tree of labeled PCs from 3-month-old mice with an LSM900 confocal microscope (63×/1.40 objective) and applied a *z*-stack scan (voxel size 0.033 × 0.033 × 0.300 µm) to ensure high 3D resolution. Manual inspection of *z*-stack images is prone to error due to the challenge of tracking fine structures across multiple *z*-planes. This limitation becomes especially pronounced in PCs, whose densely packed dendritic spines make the evaluation particularly time-consuming and susceptible to human error. To overcome this challenge, we used Imaris, a machine learning-based image analysis software (Swanger et al., 2011), to quantify PC dendritic spines. Acquired images were 3D projected and deconvoluted for PSF using Imaris (Fig. 4*A*). With the Filament Tracer in the Imaris software, we reconstructed the dendritic segments and spines into 3D meshes (Fig. 4*B*). We then statistically analyzed differences in spine properties between PCs from the anterior and posterior vermis. To avoid inflation of statistical power from hundreds of spines within the same cell, the average of measurements from all reconstructed spines from each PC was used. LMM with “MouseID” included as a subject-level random effect was used to further account for pseudoreplication. We detected a significantly higher spine density in PCs of the anterior vermis (Fig. 4*C*). The spine length in PCs from both anterior and posterior vermis does not significantly differ (Fig. 4*D*). The dendritic spines of PCs from the anterior vermis exhibited a significantly smaller head diameter. These results revealed by our sparse AAV-mediated labeling and analytical pipeline suggest that PCs between the anterior and posterior vermis in mice may possess different features, such as density and geometry of their dendritic spines.

### Altered PC morphology in a mouse model of DS

**Figure 5.**
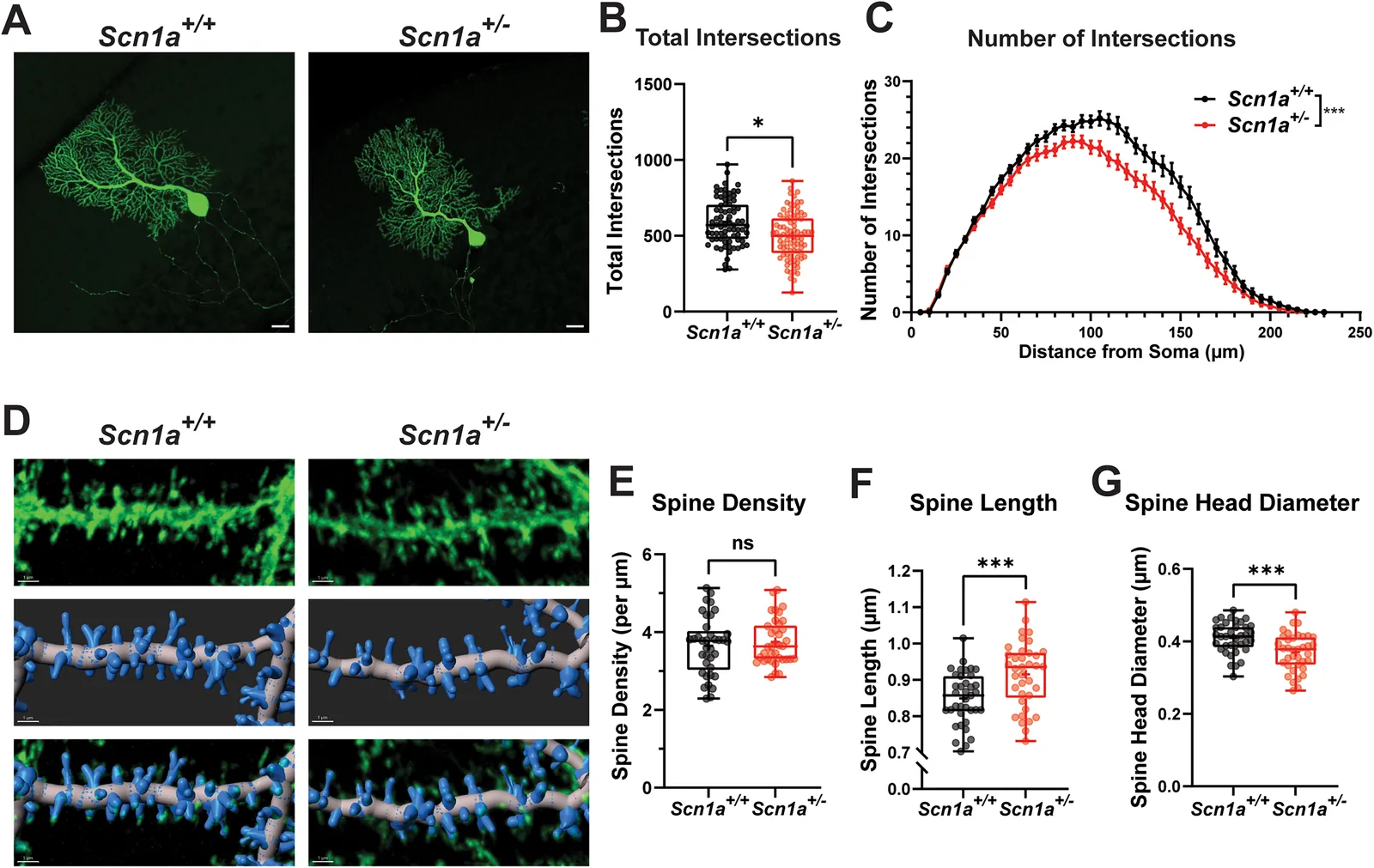
Quantification of PC morphology in a mouse model of DS. ***A***, Representative fluorescence image of labeled PCs from *Scn1a^+/−^* and control animal (*Scn1a^+/+^*). Scale bars, 20 µm. ***B***, Statistical analysis revealed a significant decrease in the total intersections in PCs from *Scn1a^+/−^* mice. *N* = 76 PCs from four *Scn1a^+/+^* mice (2 aged P16, 2 aged P19) and *N* = 85 PCs from *Scn1a^+/−^* mice (2 aged P16, 2 aged P19). LMM, *F*_(1, 5.742)_ = 8.002; *p* = 0.031, “MouseID” as a subject-level random effect. ***C***, Sholl analysis of the PC dendritic structure revealed a significant difference in PCs from *Scn1a^+/−^* mice compared with *Scn1a^+/+^* controls. *N* = 76 PCs from 4 *Scn1a^+/+^* mice (2 aged P16, 2 aged P19) and *N* = 85 PCs from *Scn1a^+/−^* mice (2 aged P16, 2 aged P19). GLMM (negative binomial, log link, “MouseID” as a subject-level random effect): radius (*F*_(43,7314)_ = 111.006; *p* < 0.001), genotype (*F*_(1,7314)_ = 38.854; *p* < 0.001), genotype by radius interaction (*F*_(42,7314)_ = 1.329; *p* = 0.076). ***D***, Representative fluorescence image and reconstructed 3D mesh of the dendritic spines from *Scn1a^+/−^* mice and *Scn1a^+/+^* controls. Scale bars, 1 µm. ***E***, Quantification and statistical analysis of the spine density in PCs from *Scn1a^+/−^* mice and *Scn1a^+/+^* controls did not detect a difference. *N* = 37 PCs from four *Scn1a^+/+^* mice (2 aged P16, 2 aged P19) and *N* = 37 PCs from *Scn1a^+/−^* mice (2 aged P16, 2 aged P19). LMM, *F*_(1,72)_ = 0.388; *p* = 0.535, “MouseID” as a subject-level random effect. ***F***, Quantification and statistical analysis of the spine length revealed a significant increase in PCs from *Scn1a^+/−^* mice compared with *Scn1a^+/+^* controls. *N* = 37 PCs from 4 *Scn1a^+/+^* mice (2 aged P16, 2 aged P19) and *N* = 37 PCs from *Scn1a^+/−^* mice (2 aged P16, 2 aged P19). LMM, *F*_(1,72)_ = 12.099; *p* < 0.001, “MouseID” as a subject-level random effect. ***G***, Quantification and statistical analysis of the spine head diameter revealed a significant decrease in PCs from *Scn1a^+/−^* mice compared with *Scn1a^+/+^* controls. *N* = 37 PCs from four *Scn1a^+/+^* mice (2 aged P16, 2 aged P19) and *N* = 37 PCs from *Scn1a^+/−^* mice (2 aged P16, 2 aged P19). LMM, *F*_(1,71.025)_ = 16.810; *p* < 0.001, “MouseID” as a subject-level random effect.

The mouse model of DS (*Scn1a^+/−^*) has been demonstrated to exhibit altered synaptic function in both GABAergic interneurons and PCs (Yu et al., 2006; Kalume et al., 2007). *Scn1a* codes for the alpha subunit of a voltage-gated sodium channel. Based on these reported functional alterations, we applied our method to examine PC dendritic arborization and dendritic spine morphology in *Scn1a^+/−^* mice. PCs from the anterior and posterior lobules were pooled for analysis. We observed a reduction in the total number of dendritic intersections in the PCs from *Scn1a^+/−^* mice compared with controls (Fig. 5*A*,*B*). Sholl profile analysis further revealed significant differences in the distribution of dendritic branching across radial distances, with a shift toward reduced intersections at distal radii in the PCs from *Scn1a^+/−^* mice (Fig. 5*A*,*C*). We also observed a significant increase in spine length and a decrease in spine head diameter in *Scn1a^+/−^* PCs, while spine density remained similar to control cells (Fig. 5*D*–*G*). Altogether, these findings indicate that PCs in *Scn1a^+/−^* mice exhibit reduced dendritic complexity and spine features, consistent with an immature morphology. These results demonstrate that our method is well suited for analyzing PC morphological and synaptic pathology in disease-relevant contexts.

## Discussion

Morphological characterization of PCs is often challenging, due to the densely packed architecture and frequent overlap among neighboring neurons. To visualize the complex morphological architecture of PCs, researchers employ multiple labeling approaches, including Golgi staining, ballistic delivery of lipophilic dyes, immunohistochemical labeling, conditional transgenic mouse lines, intracellular dye filling, and stereotaxic delivery of viral vectors encoding fluorescent reporters into the cerebellum (Stewart, 1978; O’Brien and Unwin, 2006; Kaneko et al., 2011; Louis et al., 2014; Sugawara et al., 2017; Sakai et al., 2019). However, labor-intensive and technically demanding protocols, together with the inherent structural overlap of PCs, remain major challenges across these approaches (Rosoklija et al., 2003; Klepukov and Apryshko, 2019; Cauzzo et al., 2024).

In this study, we present an AAV-mediated, Cre-dependent sparse PC labeling strategy as an improved alternative approach. To achieve sparse PC labeling, we performed intracerebroventricular injection of diluted AAV*-pCAG-FLEX-EGFP-WPRE* into *BAC-Pcp2-IRES-Cre* mouse pups. Neonatal intracerebroventricular delivery of AAV is a simple, cost-effective, and minimally invasive approach that enables rapid recovery without inducing significant adverse effects (Chakrabarty et al., 2013; Kim et al., 2013, 2014; Ahmed et al., 2024; Yang et al., 2024). Using this delivery method, studies have shown that PC axon collaterals can be reliably labeled, enabling detailed reconstruction and analysis of their synaptic contacts (Guo et al., 2016; Witter et al., 2016). Our Cre-dependent, conditional AAV approach enabled single-cell–level labeling of PCs without inducing mechanical damage to the cerebellar tissue, yielding well-resolved dendritic arbors and clearly defined dendritic spines across multiple developmental stages. The high signal-to-noise ratio and excellent structural clarity of the acquired images facilitate accurate morphological characterization without requiring complex image processing procedures, such as segmentation.

Following sparse labeling of PCs, we introduced and refined two analytical pipelines aiming to precisely quantify their morphological features: the dendritic arborization and spines. To characterize PC dendritic complexity, we preprocessed and skeletonized the PCs using CellProfiler (Carpenter et al., 2006; Stirling et al., 2021). The Sholl analysis was then carried out with Fiji (Schindelin et al., 2012; Ferreira et al., 2014). Both CellProfiler and Fiji are open-source image analysis software broadly used for morphological analysis. The preprocessing and skeletonization in CellProfiler are semiautomatic and enable high-throughput analysis. To characterize PC dendritic spines, we 3D reconstructed the spines through Imaris (Swanger et al., 2011). Further measurements of spine were carried out automatically by Imaris after 3D reconstruction. This pipeline provides a more efficient and accurate alternative compared with classical manual quantification.

To demonstrate the potential and utility of our approach, we first applied it in a biological context to examine the dendritic complexity and spines between PCs from the anterior and posterior vermis. We did not find a significant regional difference in PC dendritic complexity in mice at 1, 3, and 6 months of age. However, we identified a shift in branching patterns between PCs from the anterior and posterior lobules in 1- and 6-month-old mice. Such moderate morphological differences may correspond to the underlying transcriptional and physiological variations between PCs from the anterior and posterior lobules, as reported in previous studies (Zhou et al., 2014; Wu et al., 2019; Beekhof et al., 2021; Chen et al., 2022). Furthermore, we revealed some differences in the density and geometry of dendritic spines from PCs between the anterior and posterior vermis. These results are consistent with previous studies reporting that subtypes of PCs exhibit distinct physiological properties and may differentially enrich in the anterior and posterior vermis of the cerebellum (Wadiche and Jahr, 2005; Zhou et al., 2014; Chen et al., 2022).

Having established the utility of our approach in a biological context, we next applied it to a pathological context using *Scn1a^+/−^* mice, a disease model of DS. Given that neuronal activity is a key regulator of dendritic branching and spine development, we examined PC morphology in *Scn1a^+/−^* mice. We found dendritic atrophy and dendritic spine immaturity in *Scn1a^+/−^* PCs, consistent with altered firing properties. Overall, we demonstrated that this sparse labeling method is suitable for both conventional 2D analyses of dendritic morphology and fine-scale 3D characterization of dendritic spines under physiological and pathological conditions.

Compared with the classical cell labeling approaches used for characterizing dendritic arbor and spine of PCs, our method offers several potential opportunities. First, neonatal intracerebroventricular injection bypasses the need for stereotaxic surgery and offers a relatively straightforward, time-efficient, and minimally invasive procedure. Second, through Cre-dependent GFP expression, the labeling is restricted to PCs and sparse enough to achieve single-cell resolution. It supports imaging and analysis throughout the mouse lifespan. Third, the cerebellar slices require no additional incubation with dyes, antibodies, or hazardous chemicals. Finally, the tissue samples are fixed and therefore do not require preserving live tissues and utilizing specialized equipment and techniques, which are typically essential during the microinjection of fluorescence dyes. Our approach circumvents common challenges associated with acute brain slicing, including dendritic spine degradation and neuronal degeneration or loss. We acknowledge that combining several existing genetic and viral strategies, including cell-type–specific promoters, Supernova-based single-cell labeling and membrane-targeting approaches such as the CAAX motif can achieve high-quality neuronal labeling and visualization of fine cellular structures (Kim et al., 2015; Luo et al., 2016; Nitta et al., 2017; Ning et al., 2022). In comparison, our workflow does not rely on specialized constructs or cell-type–specific redesign of vectors. It is straightforward to implement and achieves sparse labeling through titration of a standard Cre-dependent viral system. Moreover, the strategy of titrating *AAV-FLEX-EGFP* to achieve sparse single-cell labeling can be readily extended to other Cre driver lines. Furthermore, the cell labeling workflow is flexible and can be extended to accommodate more complex experimental paradigms. As an example, PC-labeled cerebellar slices can be processed with immunofluorescence procedures to stain for PC subtype-specific markers, such as PLCB4 and ALDOC. Although these PC subtypes are enriched in either the anterior or posterior vermis, their distributions overlap. Combining sparse labeling with subtype marker staining could therefore distinguish region-specific from subtype-specific morphological differences.

Despite the strength of our approach, several limitations should be considered. To enable PC-specific labeling, our approach requires a PC-specific transgenic Cre mouse line. Compared with other approaches directly applicable to wild-type mice, maintaining a transgenic strain can be costly and time-consuming. The labeled PCs are not regionally specific compared with microinjection. Multiple cerebellar slices may need to be screened to obtain labeled PCs in the desired region. Finally, the successful implementation of our method requires some technical knowledge and training.

In conclusion, we introduced a streamlined sparse labeling method for cerebellar PCs, along with analytical pipelines for assessing dendritic complexity and spine morphology. Using a simple experimental setup, we demonstrated the applicability of our method for quantitative morphological studies in both physiological and pathological contexts. By integrating complementary approaches, including electrophysiological recording, expansion microscopy, super-resolution microscopy, and whole-brain clarity (Chung et al., 2013; Renier et al., 2014; Chen et al., 2015), our method and analytical pipelines could be applied to investigate diverse aspects of PC biology in cerebellar development, function, plasticity, and disease.

## Synthesis

Reviewing Editor: David Schoppik, New York University Grossman School of Medicine Decisions are customarily a result of the Reviewing Editor and the peer reviewers coming together and discussing their recommendations until a consensus is reached. When revisions are invited, a fact-based synthesis statement explaining their decision and outlining what is needed to prepare a revision will be listed below. The following reviewer(s) agreed to reveal their identity: Pawel Switonski, Hiroyuki Hioki.

1) The term "high-resolution" appears repeatedly throughout the manuscript, but its precise meaning remains unclear. Please clarify in what sense this term is being used.

2) The authors state that they provide "an easily adaptable workflow that is efficient, robust, and well-suited for examining PC morphology during cerebellar development and related pathogenic conditions" (lines 37-39). Please explicitly explain how this workflow improves upon, or is expected to improve upon, previous analytical methods.

3) The authors write that they employed a mouse model of Dravet syndrome to demonstrate the utility of this approach for analyzing PC morphology in a disease-associated state (lines 113-115). Please explain why this model was selected among the many available disease models.

4) Figure 2 requires substantial revision. In its current form, the results are presented in a rather descriptive manner, and the main message is difficult to grasp. Please show only representative images and to include quantitative graphs, such as the number of infected cells or the distribution of labeled cells, and move the remaining images to the Extended Data. We believe this will allow the main figure to more effectively convey the key message while still providing complete documentation of the dataset.

5) The orientation of Purkinje cells should be standardized across figures as much as possible. For example, the cell bodies should preferably be positioned consistently at the bottom of the images.

6) In Fig.5A, the images after skeletonization and after Sholl analysis are almost entirely black and difficult to interpret. Please replace them with clearer images.

7) The detection sensitivity and specificity of Imaris Filament Tracer should be carefully validated. For example, in the "Posterior Lobules" image in Fig.4B, the horizontally oriented dendrite at the top appears to be partially missing from the tracing. Similar concerns apply to spine detection. Based on Fig.4 and 5, some structures seem not to have been detected. Because this issue could affect the quantitative results, the authors should carefully confirm the reliability of the tracing and detection procedures.

## Author Response

"A Streamlined Workflow for Purkinje Cell Labeling and High-Resolution Analysis of Dendrite and Spine in Mice" (eN-MNT-0455-25).

Dear Dr. Schoppik, Thank you for your time and for considering our revised manuscript. We sincerely appreciate your thoughtful coordination of the review process and your clear synthesis of the reviewers' comments, which greatly facilitated our revisions and made the revision process much more straightforward.

Please find below our detailed point-by-point responses to the reviewers' comments. We hope that our responses and the revised manuscript adequately address the reviewers' and editor's comments and that the manuscript will now be considered suitable for publication in eNeuro.

Best regards, Yang ------------------------------------------------------------------------------------------------------------ Point-to-point response to reviewer comments Synthesis Statement for Author (Required): (1) The term "high-resolution" appears repeatedly throughout the manuscript, but its precise meaning remains unclear. Please clarify in what sense this term is being used.

Response: We thank the reviewer for this valuable comment. In this context, "high-resolution" refers to the ability of our labeling approach to visualize fine Purkinje cell structures, including dendritic arbors and spines, when combined with high-resolution imaging modalities such as confocal microscopy. To address reviewer's comments, we have revised the Significant Statement to clarify this meaning: (lines 45-47) "Obtaining specific labeling of cerebellar PCs in vivo that is compatible with high-resolution imaging and straightforward analysis of dendritic structure and fine spine features remains challenging." (2) The authors state that they provide "an easily adaptable workflow that is efficient, robust, and well-suited for examining PC morphology during cerebellar development and related pathogenic conditions" (lines 37-39). Please explicitly explain how this workflow improves upon, or is expected to improve upon, previous analytical methods.

Response: We have revised the text to clarify that the main contribution of this study is not a fundamentally new analytical method, but rather an improved labeling method that supports established analyses of PC morphology. Specifically, this method enables sparse and specific visualization of individual cerebellar PCs, thereby facilitating downstream morphological assessments such as dendritic arbor analysis and examination of fine features of spines. Thus, the expected improvement over previous analytical practices lie not in replacing existing analytical methods, but in providing high-quality labeled material that makes conventional morphological analyses more straightforward and practical for studying PC development and related pathogenic conditions.

We have revised the sentence: (lines 37-38) "Thus, we provide a labeling method that is easily adaptable, efficient, robust, and well suited for examining PC morphology during cerebellar development and related pathogenic conditions." (3) The authors write that they employed a mouse model of Dravet syndrome to demonstrate the utility of this approach for analyzing PC morphology in a disease-associated state (lines 114-116). Please explain why this model was selected among the many available disease models.

Response: We thank the reviewer for this comment. The Dravet syndrome mouse model was selected as a disease-associated model because it is a well-established neurodevelopmental disease model with reported motor abnormalities and altered PC firing, making it relevant for evaluating PC morphology. Our purpose was not to compare multiple disease models or to claim that this model is uniquely suited for PC morphological analysis. We used this model, as it was readily available and had been established in our laboratory. (4) Figure 2 requires substantial revision. In its current form, the results are presented in a rather descriptive manner, and the main message is difficult to grasp. Please show only representative images and to include quantitative graphs, such as the number of infected cells or the distribution of labeled cells, and move the remaining images to the Extended Data. We believe this will allow the main figure to more effectively convey the key message while still providing complete documentation of the dataset.

Response: We are very grateful to the reviewer for pointing this out. We have revised Figure 2 to include representative images and quantitative graph. The complete image set has been moved to Extended Figure 2-1 in the revised manuscript. (5) The orientation of Purkinje cells should be standardized across figures as much as possible. For example, the cell bodies should preferably be positioned consistently at the bottom of the images.

Response: We have re-oriented the PC images, where possible, so that the cell bodies are positioned consistently at the bottom of the images, and the cells face a similar direction across all figures. Specifically, these include Figures 1C, 3A, 3B, 3E, 3H, 5A, and 5B. (6) In Fig.5A, the images after skeletonization and after Sholl analysis are almost entirely black and difficult to interpret. Please replace them with clearer images.

Response: We thank the reviewer for this comment. We have replaced the skeletonization and Sholl analysis images in Fig. 5A with clearer images to enhance their visibility and improve data interpretation. (7) The detection sensitivity and specificity of Imaris Filament Tracer should be carefully validated. For example, in the "Posterior Lobules" image in Fig.4B, the horizontally oriented dendrite at the top appears to be partially missing from the tracing. Similar concerns apply to spine detection. Based on Fig.4 and 5, some structures seem not to have been detected. Because this issue could affect the quantitative results, the authors should carefully confirm the reliability of the tracing and detection procedures.

Response: We thank the reviewer for this important comment. We agree that the reliability of dendritic tracing and spine detection is critical for accurate quantitative analysis. We have carefully re-examined the tracing and spine detection results in Fig.4 and 5.

For dendritic tracing, some dendritic branches that appear visible in the representative images were intentionally excluded from the analysis rather than missed by the tracing procedure. Specifically, to avoid ambiguity caused by overlapping or crossing neurites, we restricted the regions of interest to dendritic branches of tertiary or higher order that did not contain crossing neurites. This criterion was applied consistently across all samples.

For spine detection, we agree that some structures may appear undetected in the representative images, which are shown in orthogonal views. However, because spine assignment depends on the 3D relationship between the protrusion and the analyzed dendritic segment, all spine detections were inspected in the 3D view. In some cases, structures that appeared to be spines in a single view were found, after rotation in 3D, to belong to neighboring dendrites or to be disconnected from the analyzed dendritic segment. These structures were therefore excluded.

We also acknowledge that automated detection may not capture every structure perfectly. To minimize potential bias, dendritic tracing and spine detection parameters were optimized using representative images and then applied consistently across all samples. Automated reconstruction and detection results were subsequently subjected to careful post hoc visual inspection and quality-control correction before quantification. In addition, values from all detected spines within each cell were averaged, and the cell-level mean was used for statistical analysis. To account for individual differences among mice, we used a linear mixed model with mouse identity included as a random effect.

Together, these standardized detection, 3D verification, post hoc quality-control, and statistical procedures were used to minimize potential detection bias and support the reliability of the quantitative analysis.

In the revised manuscript, we have revised the Methods to better clarify these procedures: (lines 223-227) "The primary and secondary dendritic branches of PC are largely free of spines. Therefore, we defined the ROIs as the dendritic branches of tertiary order or higher that did not cross other neurites. Dendritic tracing and spine detection parameters were optimized using representative images and then applied consistently across all samples." (lines 234-238) "The output meshes from Imaris were carefully validated by the researcher under 3D view. Poorly reconstructed dendritic segments, ambiguous branches, and spine detections that were incorrectly assigned to the analyzed dendrite were removed during post hoc quality control before quantification." (lines 239-241) "Notably, to avoid inflated statistical power from pseudo-replication and to reduce the influence of potential detection bias, values from all detected spines within each cell were averaged, and the resulting cell-level mean was used for statistical analysis."
